# The relationship between negative psychological state and quality of life among cardiovascular disease patients in China: the masking effect of abnormal dietary behavior

**DOI:** 10.3389/fcvm.2025.1406890

**Published:** 2025-02-12

**Authors:** QingNing Chang, HaiBo Ma, Can Zhang, Xin Li, YiBo Wu, LiNa Ha

**Affiliations:** ^1^ Medical Experimental Center, General Hospital of Ningxia Medical University, Yinchuan, China; ^2^ The First School of Clinical Medicine, Ningxia Medical University, Yinchuan, China; ^3^ The Third School of Clinical Medicine, Ningxia Medical University, Yinchuan, China; ^4^ School of Public Health Management, Ningxia Medical University, Yinchuan, China; ^5^ School of Public Health, Peking University, Beijing, China; ^6^ School of Humanities and Management, Ningxia Medical University, Yinchuan, China

**Keywords:** negative psychological state, abnormal dietary behavior, quality of life, family health, cardiovascular disease

## Abstract

**Background:**

It is well known that abnormal dietary behavior increases the risk for cardiovascular disease especially if the person is depressed and/or anxious. The purpose of this study was to construct a moderated mediation model to explore the roles of abnormal dietary behavior and family health in the mechanism through which depression/anxiety influences Quality of life (QoL) in patients with cardiovascular disease.

**Methods:**

A field survey was conducted in China and ultimately included 730 patients with cardiovascular disease aged 20–60 years. Data were collected using the Europe Quality of five-dimensional five-level questionnaire, Short-Form of the Eating Behavior Scale, Patient Health Questionnaire-9, Generalized Anxiety Disorder-7, and the Chinese version of the short-form of the Family Health Scale. All data were analyzed using SPSS Statistics 23.0.

**Results:**

(1) Depression was negatively associated with QoL (*r* = −0.386/−0.230, *p* < 0.001), and was positively correlated with abnormal dietary behavior (*r* = 0.377, *p* < 0.001). Anxiety was negatively associated with QoL (*r* = −0.383/−0.231, *p* < 0.001), and was positively correlated with abnormal dietary behavior (*r* = 0.333, *p* < 0.001). Abnormal dietary behavior was negatively correlated with QoL (*r* = −0.077/−0.119, *p* = 0.039/0.001). (2) In the mediation model, abnormal dietary behavior only had a masking effect on the relationship between depression and QoL, with a mediating effect size of 7.18%. The mediating effect of abnormal dietary behavior between anxiety and QoL was not significant. (3) The mediating effect size of abnormal dietary behavior between depression/anxiety and QoL increased to 14.77% and 13.57% in unhealthy families. The above masking mediation effect was not significant in healthy families.

**Conclusions:**

Abnormal dietary behavior positively mediated the relationship between depression and QoL and attenuated the negative effect of depression on QoL in patients with cardiovascular disease. The masking mediating effect of abnormal dietary behavior between depression/anxiety and QoL was stronger for patients in unhealthy families.

## 1 Introduction

Cardiovascular diseases (CVD) is a group of primary diseases of the heart and vasculature and arising as a co-morbidity with numerous pathologies (1). In recent years, the incidence rate of this disease is increasing and the onset age is getting lower, which is a serious threat to human health (2). The number of patients with CVD in China has reached 330 million, ranking first in the world in terms of mortality (3). Prolonged courses, unfavorable prognosis, propensity for recurrence and substantial financial burden associated with this disease contribute to diminished Quality of life (QoL) among patients (4), which may further increase disease mortality.

The World Health Organization (WHO) defines QoL as people's sense and experience of their physical state, mental function, social ability and overall personal condition based on their socioeconomic, cultural background and value orientation. To improve outcomes in cardiovascular patients, improving their QoL must go hand in hand with cardiovascular medication (5). Thus, QoL is considered one of the main goals in the prevention and treatment of cardiovascular disease, which can help review and identify medical decisions for each individual.

Research had shown a strong correlation between cardiovascular disease and psychological state (6). The incidence of negative psychological states such as depression and anxiety was significantly higher in patients with CVD than in the general population (7). Some clinical studies had found that negative mental states may aggravate the condition and affect patients' QoL (8, 9). This is because mental states stimulate sympathetic nerve activity, which creates fluctuations in the cardiovascular system such as tachycardia, hypertension, and blood flow reorientation (10). In the treatment of hypertensive patients, medications should be used in conjunction with some complementary and alternative therapies such as yoga and meditation. Research had found that these alternative therapies could reduce the side effects of depression and anxiety in patients with CVD, thereby improving their QoL and achieving the desired antihypertensive effect (11). Therefore, this study proposes the hypothesis:

*Hypothesis 1 (H1)*: depression/anxiety is negatively related to QoL of cardiovascular patients.

Negative psychological states (such as depression/anxiety) are associated with an increased risk of abnormal dietary behavior, which may affect physical health, including cardiovascular diseases (12, 13). Psychophysiological studies have reported that individuals with disordered dietary behavior exhibit atypical cardiovascular reactions to acute psyche (14). According to some studies, people with negative psychology tend to develop unhealthy eating habits, consume food with a high content of fat and sugar, or exhibit anorexia nervosa (15, 16). Conversely, optimism is associated with positive health outcomes, healthier dietary behaviors, higher overall diet quality and a reduced risk of cardiovascular disease (17). Therefore, we proposed the following hypothesis:

*Hypothesis 2 (H2)*: negative psychological state (depression and anxiety) is positively related to abnormal dietary behavior.

A healthy diet can improve QoL in individuals with cardiovascular diseases (18). Studies have shown that a diet rich in fruits and vegetables has a significant protective effect on the heart (19). Many high concentrations of bioactive compounds are present in fruits and vegetables, such as unsaturated fatty acids, polyphenols, fiber, and other ingredients, can play antioxidant, anti-inflammatory, and anti-thrombotic roles, which help to delay the occurrence and progression of cardiovascular disease (20). For example, a Mediterranean diet may improve the QoL of individuals with cardiovascular diseases by improving disease-related physiological indices (21). Patients with high Mediterranean diet scores have reported higher QoL scores (22). On the contrary, high-salt and high-fat diet could cause oxidative stress damage to the myocardium and may even induce inflammation (23). Research had found that the inflammatory potential of unhealthy diets could lead to the development of chronic diseases and reduce the QoL for patients (24). In addition, irregular eating behaviors may contribute to the occurrence of cardiovascular diseases due to circadian rhythm imbalances in the body (25). Thus, we proposed the following hypothesis:

*Hypothesis 3 (H3)*: abnormal dietary behavior is negatively correlated with QoL in patients with CVD.

Family health is a resource at the family unit level that develops from the intersection of the health of each family member and their interactions and capacities as well as the family's internal and external resources (26). Healthy families have excellent family functions, indicating that family dynamics are balanced and harmonious. Family members have the ability to cope with difficulties, resolve conflicts, and assign roles that can simultaneously support individual physical, psychological and social development (27). There is growing evidence that engaging family members in care improves the care experience and achieves better outcomes for cardiovascular patients (28). A well-functioning family unit can enhance self-management skills, thereby guiding patients to cope effectively with negative emotions and ultimately improve their QoL (29). Consequently, we proposed the following hypotheses:

*Hypothesis 4 (H4)*: family health moderates the process through which dietary behaviors mediates the effect of depression/anxiety on QoL.

QoL is an important outcome indicator for evaluating the intervention and treatment effects of patients with CVD (30, 31). However, little is currently known about the association between cardiovascular diseases and QoL (32). Depression and anxiety are frequently comorbid in patients with CVD and associated with poor QoL (33). A study had found that BMI alone significantly accounted for 11.8% of the variability in QoL and increased with the addition of anxiety and depressive symptoms (34). BMI is related to obesity, dietary behavior, and other factors. However, research on the interaction between depression/anxiety, dietary behavior and QoL is limited. Family health is important in the management of CVD, but few studies have examined family health and QoL in Patients with CVD (35). The aim of this study is to develop a moderated mediating effect model to assess the mediating effect of dietary behavior between depression/anxiety and QoL and the moderating effect of family health in patients with CVD. This will provide a theoretical basis for the “psycho-cardiology” population QoL improvement model.

## 2 Methods

### 2.1 Participants

The survey was conducted from May 2021 to September 2021 using a multistage sampling method (36, 37). The provincial capitals of 23 provinces, 5 autonomous regions, and 4 municipalities directly under the Central Government (Beijing, Tianjin, Shanghai, and Chongqing) in China were included. Non-provincial cities of provinces and autonomous regions were selected using a random number table. Ultimately, 120 cities were included in this study. Investigators or teams (≤10 people) were recruited in these cities to collect the questionnaires. According to quota attributes (such as gender, age, urban, and rural distribution), residents in these cities were selected by quota sampling. The population distribution of the obtained samples was basically consistent with the population characteristics of the “results of the seventh National Population Census in 2021”. This study was ethically reviewed (JNUKY-2021-018). All methods were performed in accordance with the relevant guidelines and regulations. Ultimately, 730 patients were selected for further analysis. Inclusion criteria were: (1) participants aged between 20 and 60 years (The Eating Behavior Scale was inapplicable to residents less than 20 or more than 60 years old); (2) any kind of cardiovascular disease diagnosed: dyslipidemia, hypertension, coronary heart disease, and so on; (3) voluntary participation and filling in the informed consent form; (4) participants who could complete the questionnaire survey independently or with the help of investigators; and (5) participants answered the questionnaire after carefully reading and understanding the meaning of each item in the questionnaire. The exclusion criteria were as follows: (1) participation in other similar research projects; (2) response time was too short (≤1,200 s) or too long (≥3,000 s); (3) incomplete questionnaire content; (4) the answer was clearly unreasonable; (5) the logic of the questions before and after the questionnaire was inconsistent; and (6) the answer options were all the same or had a pattern.

### 2.2 Measures

#### 2.2.1 The general information questionnaire

Information on demographic, anthropometric, and medical history parameters such as gender, age, ethnicity, permanent residence, monthly per capita household income, highest educational level, current confirmed disease type, height, and weight were collected. Subsequently, height and weight were used to calculate the Body Mass Index (BMI). Based on the confirmed diseases, participants were classified into the following four categories: (1) only one cardiovascular disease; (2) diagnosis of two or more cardiovascular diseases; (3) cardiovascular disease with mood disorders; (4) cardiovascular disease combined with other diseases.

#### 2.2.2 The Europe quality of five-dimensional five-level questionnaire (EQ-5D-5L)

The EQ-5D-5L was used to evaluate health-related QoL of the population. The EQ-5D-5L descriptive system includes the following five dimensions: mobility, self-care, anxiety or depression, daily activities, pain or discomfort. Each dimension was articulated into five severity levels: 1 = no problems, 2 = slight problems, 3 = moderate problems, 4 = severe problems and 5 = extreme problems (or unable to). Consequently, 3,125 (5^5^) possible health states were determined by a combination of responses and identified with a unique five-digit number. The states “11111” means there are no problems in any dimension, while the states “55555” means that there are serious problems in all five dimensions. Each health state can be converted into a single index value by using predefined preference weights collected at the population level. The Chinese EQ-5D-5L index ranges from −0.391 for “55555” and 1.000 for the healthiest state (“11111”) (38). The EQ-5D questionnaire also includes a visual analogue scale (EQ-VAS). Participants rated their current health status on an EQ-VAS ranging from 0 to 1, obtained by dividing the number marked on the scale by 100. Compared with those with low scores, respondents who obtained high utility scores or VAS scores had higher QoL.

#### 2.2.3 The short-form of the eating behavior scale (EBS-SF)

The EBS-SF is a short version of the 30-item Sakata Eating Behavior Scale (EBS), which evaluates dietary behavior abnormalities (39). The EBS-SF scale mainly includes 7 items, that reflect 7 areas of eating rhythm abnormalities (Eat at all different times), feeling of satiety (Do not feel satisfied unless I eat until full), eating style (Eat fast), cognition of constitution (Tend to gain weight more easily than others), meal contents (Like oily foods), substitute eating and drinking (Eat if others around me are eating) and motivation for eating (When buying food, I am not content unless I buy more than necessary). Responses were chosen from 4 options: “1 = strongly disagree”, “2 = somewhat disagree”, “3 = somewhat agree”, and “4 = strongly agree”. The total scores were calculated to be between 7 and 28. Higher scores indicate abnormal dietary behaviors. In this study, Cronbach's *α* of the EBS-SF was 0.831.

#### 2.2.4 The patient health questionaire-9 (PHQ-9)

The PHQ-9 is one of the most reliable screening tool for identifying depression (40). It consists of 9 items rated on a scale ranging from “0 = not at all” to “3 = nearly every day”. The total scale score was calculated to assess depression: 0–4: Normal (no depressive symptoms or extremely mild depressive symptoms), 5–9: mild, 10–14: moderate, 15–19: moderately severe, and ≥20: severe (41). The Cronbach's *α* of the PHQ-9 was 0.924 in this study, indicating excellent internal consistency.

#### 2.2.5 The generalized anxiety disorder-7 (GAD-7)

Anxiety was assessed through GAD-7, which is a practical self-report anxiety questionnaire to describe the feelings of the past two weeks (42). There are 7-item in the scale, and assess anxiety by calculating the total score. Each item is scored from 0 to 3, and the total score ranged from 0 to 21. A higher score indicates a higher level of anxiety. The Cronbach's *α* of the GAD-7 was 0.938 in this study, indicating excellent internal consistency.

#### 2.2.6 The Chinese version of short-form of family health scale (FHS-SF)

The FHS-SF can be used to measure family health function and contain four dimensions of family social and emotional health process, family healthy lifestyle, family health resources, and family external social support (43). A total of 10 items are included, and each item adopts 5-point Likert scoring method. Entries scoring <4 points are coded as 0, scores ≥4 points are coded as 1, and the total score of the scale is 10 points. Higher scores mean higher family health. When discussing the regulating effect of family health, families scoring 0–5 points are categorized as unhealthy, and those scoring 6 points and above are classified as healthy (44). The Cronbach's *α* was 0.843 in this study.

### 2.3 Statistical analysis

Data were analyzed using SPSS Statistics 23.0, and a two-sided *p* < 0.05 was considered statistically significant. The counting data were expressed as *N* (%), and the metering data were expressed as *Mean* ± *SD*. The Harman single-factor test was used to analyze all variables in the questionnaire, revealing that there were six factors with characteristic roots exceeding 1. The first principal component could explain 30.784%, which was lower than the critical value (40%) (45). This indicated that there was no common method bias among the measured variables. The *T*-test or *F*-test was used to compare the differences in QoL scores (EQ-5D-5L and EQ-VAS) for different demographic information. Pearson's correlation analysis was used to describe the relationships among the main variables. Depression, anxiety, dietary behavior and family health were adapted as independent variables to explore their predictive role in various dimensions of QoL using regression analysis. Eventually, the utility index values of EQ-5D-5L, which were included in the model equation as dependent variables, were used to measure the QoL of the subjects. In this study, the process plug-in developed by Hayes's research model was used to test the hypothesis model further (46). Model 4 was used to verify the mediating role of abnormal dietary behavior (mediator variable) between depression/anxiety (independent variable) and QoL (dependent variable). Subsequently, we observed whether the confidence interval (CI) for each path included zero. Similarly, PROCESS macro was used to test the moderating effects of family health. Subjects were divided into healthy family functioning group/unhealthy family functioning groups, and the mediating effect of abnormal dietary behavior between depression/anxiety and QoL in different family health was assessed.

## 3 Results

### 3.1 The statistical description of the QoL scored and influencing factors

The sample consisted of 454 males and 276 females, and 550 of the participants lived in urban areas. In the EQ-5D-5L, there were significant differences in monthly per capita household income (*F* = 2.733, *p* = 0.028), current confirmed disease type (*F* = 7.055, *p* < 0.001), depression (*F* = 34.508, *p* < 0.001), anxiety (*F* = 42.548, *p* < 0.001), and family health (*t* = 31.787, *p* < 0.001). There were significant differences about EQ-VAS mean in highest education level (*F* = 3.485, *p* = 0.016), monthly per capita household income (*F* = 5.663, *p* < 0.001), current confirmed disease type (*F* = 6.623, *p* < 0.001), depression (*F* = 10.802, *p* < 0.001), anxiety (*F* = 14.192, *p* < 0.001), and family health (*t* = 22.165, *p* < 0.001). The patients with CVD and emotional disorder score their own QoL far lower than other types of patients, which may be related to the subjective state. Neither EQ-5D-5L mean nor EQ-VAS mean showed significant differences by gender, residence and BMI see Table 1.

**Table 1:** Comparison of differences in QoL among different groups.

Variable		*N* (%)	EQ-5D-5L mean	EQ-VAS mean
Gender	Male	454 (62.19)	0.91	77.91
	Female	276 (37.81)	0.92	77.59
	*T*		−0.874	0.245
Ethnicity	Han	682 (93.42)	0.92	77.89
	Minorities	48 (6.58)	0.93	76.35
	*T*		−0.599	0.619
Residence	Urban	550 (75.34)	0.92	78.74
	Rural	180 (24.66)	0.92	74.87
	*T*		0.046	2.733
Educational level	Never go to school	27 (3.70)	0.88	71.78
	Primary school	66 (9.04)	0.92	73.36
	Middle school	292 (40.00)	0.93	77.81
	University and above	345 (47.26)	0.91	79.09
	*F*		1.183	3.485*
Monthly per capita household income	0–1,500	62 (8.50)	0.87	69.26
	1,501–4,500	283 (38.77)	0.91	77.59
	4,501–7,500	237 (32.47)	0.94	78.27
	7,501–10,500	85 (11.64)	0.93	80.86
	10,501 and above	63 (8.63)	0.91	81.13
	*F*		2.733*	5.663***
BMI	≤18.4	58 (7.95)	0.91	76.98
	≥18.5 and ≤23.9	314 (43.01)	0.92	76.64
	≥24 and ≤27.9	281 (38.49)	0.92	79.02
	≥28	77 (10.55)	0.92	78.55
	*F*		0.970	1.118
Current confirmed disease type	Only one CVD	488 (66.85)	0.93	79.45
	Two or more CVD	70 (9.59)	0.92	77.77
	CVD and MD	5 (0.68)	0.90	67.40
	CVD and others	167 (22.88)	0.87	73.24
	*F*		7.055***	6.623***
Depression	Normal	300 (41.10)	0.98	82.22
	Mild	266 (36.44)	0.93	75.99
	Moderate	88 (12.05)	0.85	73.26
	Moderately severe	55 (7.53)	0.76	71.65
	Severe	21 (2.88)	0.79	72.19
	*F*		34.508***	10.802***
Anxiety	Normal	361 (49.45)	0.96	81.73
	Mild	253 (34.66)	0.91	74.10
	Moderate	96 (13.15)	0.79	73.67
	Severe	20 (2.74)	0.78	73.10
	*F*		42.548***	14.192***
Family health	Unhealth	219 (30.00)	0.87	73.43
	Health	511 (70.00)	0.94	79.65
	*t*		31.787***	22.165***

Current confirmed disease type: (1) Only one CVD, Only one cardiovascular disease; (2) Two or more CVD, Diagnose two or more cardiovascular diseases; (3) CVD and MD, Cardiovascular disease with mood disorder; (4) CVD and others, Cardiovascular disease combined with other diseases (except mood disorder).

* *p* < 0.05.

*** *p* < 0.001.

### 3.2 The correlation analysis of each Variable

Table 2 shows that all of the key variables were significantly correlated with each other. Among them, EQ-5D-5L and EQ-VAS were significantly positively correlated (*r* = 0.252, *p* < 0.001). Depression was negatively associated with QoL (EQ-5D-5L: *r* = −0.386, *p* < 0.001; EQ-VAS: *r* = −0.230, *p* < 0.001), and was positively correlated with abnormal dietary behavior (*r* = 0.377, *p* < 0.001). Anxiety was negatively associated with QoL (EQ-5D-5L: *r* = −0.383, *p* < 0.001; EQ-VAS: *r* = −0.231, *p* < 0.001), and was positively correlated with abnormal dietary behavior (*r* = 0.333, *p* < 0.001). Abnormal dietary behavior was negatively associated with family health, EQ-5D-5L and EQ-VAS (*r* = −0.196, *p* < 0.001; *r* = −0.077, *p* = 0.039; *r* = −0.119, *p* = 0.001). Demographic variables as highest education level and monthly per capita household income were mainly related to family health and EQ-VAS (*p* < 0.05). BMI was only significantly related to family health (*r* = 0.139, *p* < 0.001).

**Table 2:** Correlation matrix among variables.

	*Mean* ± *SD*	1	2	3	4	5	6	7	8	9
1. Depression	6.85 ± 5.57	1								
2. Anxiety	5.08 ± 4.50	0.819***	1							
3. Dietary behavior	17.15 ± 4.21	0.377***	0.333***	1						
4. Family health	6.58 ± 3.20	−0.287***	−0.294***	−0.196***	1					
5. EQ-5D-5L	0.92 ± 0.16	−0.386***	−0.383***	−0.077*	0.191***	1				
6. EQ-VAS	77.79 ± 16.59	−0.230***	−0.231***	−0.119**	0.219***	0.252***	1			
7. Highest education level	3.31 ± 0.79	−0.003	0.007	−0.025	0.158***	−0.008	0.112**	1		
8. Monthly per capita household income	2.73 ± 1.06	−0.040	−0.058	0.044	0.080*	0.057	0.140***	0.329***	1	
9. BMI	23.75 ± 3.47	−0.042	−0.053	0.065	0.139***	0.040	0.049	0.047	0.035	1

* *p* < 0.05.

** *p* < 0.01.

*** *p* < 0.001.

### 3.3 The multivariate linear regression of QoL

Further investigated the predictive effect of depression, anxiety, dietary behavior and family health on QoL. Depression, anxiety, dietary behavior and family health were used as specific predictors, and QoL was used as dependent variable for linear regression analysis. As stated in the QoL scale, a higher EQ-VAS score means that people feel they are in better health. The state 1 represents no problems and the state 5 represents extreme problems in EQ-5D-5L. In Table 3, the results showed that only family health was a significant predictor of EQ-VAS (*β* = 0.824, *p* < 0.001). In the dimensions of ED-5D-5L, depression was related to all dimensions and anxiety was related to dimensions other than pain/discomfort (*p* < 0.05). Dietary behavior was significantly related to usual activities and pain/discomfort (*β* = −0.009, *p* = 0.031; *β* = −0.014, *p* = 0.043). Family health had a significant relationship with mobility, self-care (*β* = −0.018, *p* = 0.004; *β* = −0.014, *p* = 0.016).

**Table 3:** Multivariate linear regression analysis of predictors of QoL.

Dependent Variables	Predictors	*R^2^*	*β*	*SE*	*t*	95% CI	
						Lower	Upper
EQ-VAS	Depression	0.077	−0.284	0.189	−1.504	−0.654	0.087
	Anxiety		−0.370	0.230	−1.609	−0.822	0.082
	Dietary behavior		−0.073	0.152	−0.476	−0.372	0.226
	Family health		0.824	0.195	4.233***	0.442	1.206
Mobility	Depression	0.099	0.016	0.006	2.640**	0.004	0.028
	Anxiety		0.018	0.008	2.333*	0.003	0.032
	Dietary behavior		−0.009	0.005	−1.803	−0.019	0.001
	Family health		−0.018	0.006	−2.877**	−0.031	−0.006
Self-care	Depression	0.107	0.014	0.006	2.549*	0.003	0.025
	Anxiety		0.019	0.007	2.827**	0.006	0.033
	Dietary behavior		−0.008	0.005	−1.757	−0.017	0.001
	Family health		−0.014	0.006	−2.416*	−0.025	−0.003
Usual activities	Depression	0.076	0.013	0.005	2.633**	0.003	0.024
	Anxiety		0.013	0.006	2.046*	0.001	0.025
	Dietary behavior		−0.009	0.004	−2.160*	−0.017	−0.001
	Family health		−0.009	0.005	−1.690	−0.019	0.001
Pain/discomfort	Depression	0.068	0.025	0.009	2.897**	0.008	0.042
	Anxiety		0.018	0.010	1.739	−0.002	0.039
	Dietary behavior		−0.014	0.007	−2.023*	−0.028	0.000
	Family health		−0.007	0.009	−0.752	−0.024	0.011
Anxiety/depression	Depression	0.165	0.029	0.008	3.474**	0.012	0.045
	Anxiety		0.039	0.010	3.894***	0.019	0.059
	Dietary behavior		−0.013	0.007	−1.879	−0.026	0.001
	Family health		−0.009	0.009	−1.011	−0.025	0.008

* *p* < 0.05.

** *p* < 0.01.

*** *p* < 0.001.

### 3.4 The mediating effect of dietary behavior

**Figure 1:**
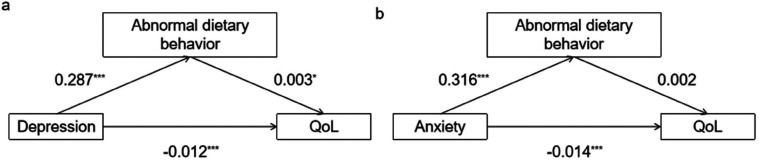
Mediating effect of abnormal dietary behavior. [**(a)** The mediating effect model between depression and QoL. **(b)** The mediating effect model between anxiety and QoL. Path coefficients correspond to unstandardized parameter estimates. *p < 0.05. **p < 0.01. ***p < 0.001.].

The mediating effect of dietary behavior (M) was further verified between depression/anxiety (X) and QoL (Y). Since the above variables other than family health have no significant predictive effect on EQ-VAS, and VAS scores are greatly affected by subjective factors. We set EQ-5D-5L as dependent variable. The Bootstrap method was adopted, and sampling was repeated 5,000 times (Table 4). The total effect of depression on QoL was significantly negative (*β* = −0.011; *p* < 0.001). Furthermore, depression significantly positively affected abnormal dietary behavior (*β* = 0.287; *p* < 0.001). Abnormal dietary behavior positively affected QoL (*β* = 0.003, *p* = 0.029). This means that the indirect effect of abnormal dietary behavior in the relationship between depression and QoL was 0.001, with a confidence interval of [0.0002, 0.002], excluding the value of 0. The direct effect of depression on QoL was also significant (*β* = −0.012, *p* < 0.001). Indirect and direct effects have opposite signs. Therefore, the conclusion is drawn that abnormal dietary behavior had a significant masking effect on the relationship between depression and QoL in cardiovascular patients (47). In other words, depressed patients improve their QoL through abnormal dietary behaviors, masking some of the negative effects of depression on QoL. The absolute value of the ratio of the reported indirect effect to the direct effect reflects the effect size of the mediating effect. The absolute value of the ratio of the mediating effect to the direct effect for abnormal dietary behavior was 7.18% (see Figure 1a). Similarly, the total effect of anxiety on QoL was significantly negative (*β* = −0.013; *p* < 0.001). Anxiety significantly positively affected abnormal dietary behavior (*β* = 0.316; *p* < 0.001). Then, abnormal dietary behavior positively affected QoL (*β* = 0.002, *p* = 0.114). The indirect effect of abnormal dietary behavior in the relationship between anxiety and QoL was 0.001, with a confidence interval of [0.000, 0.002], including the value of 0. The mediating effect of dietary behavior between anxiety and QoL is not statistically significant (see Figure 1b).

**Table 4:** Mediating effect of dietary behavior on the relationship between depression/anxiety and QoL.

Independent variable	Dependent variable	Effect	*SE*	95% CI	
				Lower	Upper
Depression	QoL	−0.011***	0.001	−0.013	−0.009
Depression	Dietary behavior	0.287***	0.026	0.234	0.336
Depression	QoL	−0.012***	0.001	−0.014	−0.010
Dietary behavior		0.003*	0.001	0.0003	0.006
Anxiety	QoL	−0.013***	0.001	−0.016	−0.011
Anxiety	Dietary behavior	0.316***	0.033	0.248	0.376
Anxiety	QoL	−0.014***	0.001	−0.017	−0.012
Dietary behavior		0.002	0.001	−0.001	0.005

* *p* < 0.05.

*** *p* < 0.001.

### 3.5 The moderating effect of family health

**Figure 2:**
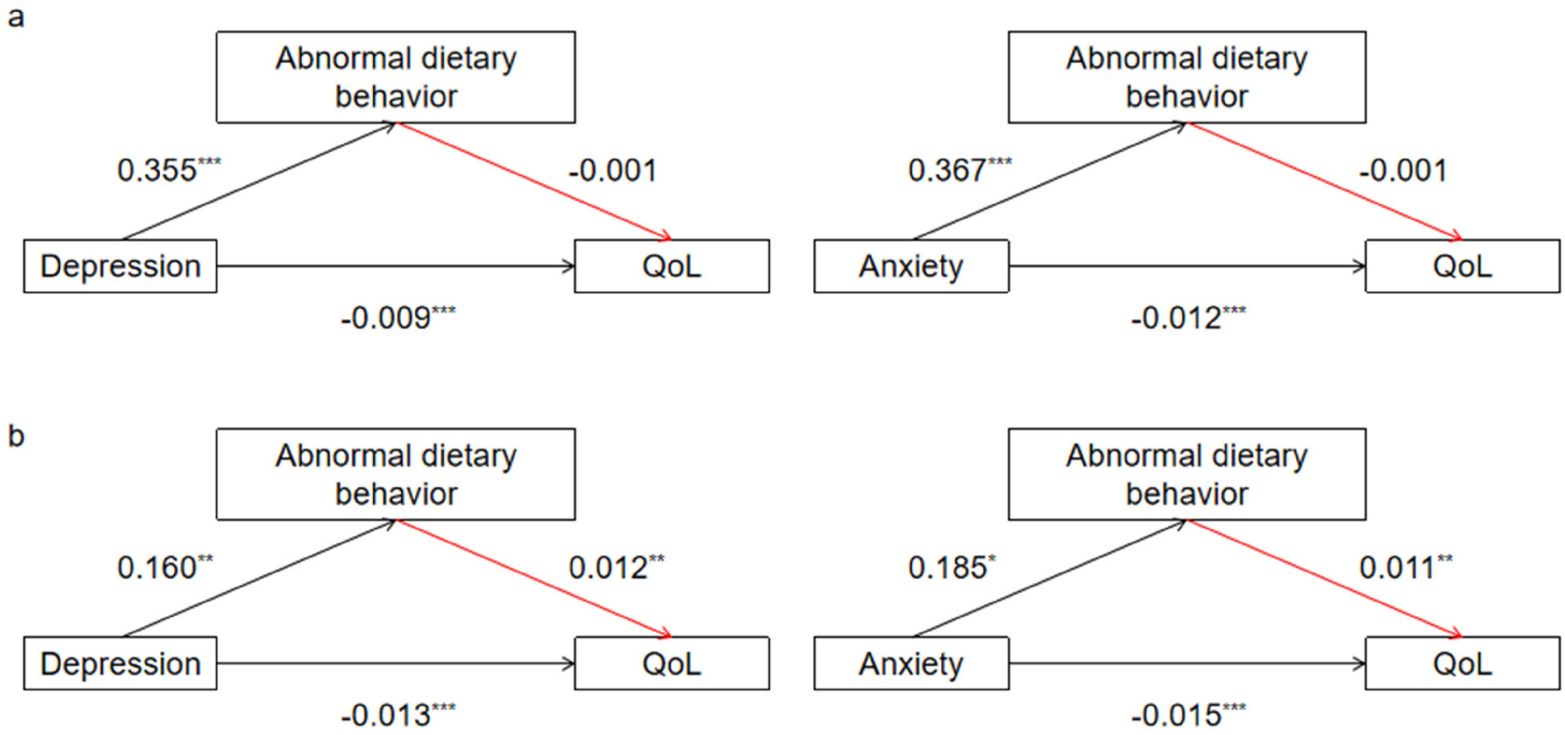
Moderating effect of family health. [**(a)** The mediating effect of abnormal dietary behavior between depression/anxiety and QoL in healthy family. **(b)** The mediating effect of abnormal dietary behavior between depression/anxiety and QoL in unhealthy family. Path coefficients correspond to unstandardized parameter estimates. *p < 0.05. **p < 0.01. ***p < 0.001.].

Abnormal dietary behavior had a partial mediating effect between depression and QoL, and abnormal dietary behavior masked the negative influence of depression on QoL. In *H4*, we predict that family health will moderate the entire mediating effect mechanism. The results were shown in Table 5. When family function is healthy, the second half path of the mediating effect, that is, the influence of abnormal dietary behavior on QoL is no longer significant (depression: *β* = −0.001, *p* = 0.668; anxiety: *β* = −0.001, *p* = 0.427) (see Figure 2a). This means that the mediating effect of dietary behavior between negative psychological state (depression/anxiety) and QoL is not statistically significant. However, in families where family function is unhealthy, each path coefficient of the mediating effect was significant. The indirect effect was opposite to the direct effect sign, while the masking effect of abnormal dietary behavior was stronger. The mediating effect size of dietary behavior between depression and QoL increased from 7.18% to |0.160*0.012/−0.013| = 14.77%. The mediating effect size of dietary behavior between anxiety and QoL was |0.185*0.011/−0.015| = 13.57%. This indicated that family health enhances the masking mediating effect of dietary behavior. The reason why the indirect effect and the direct effect had opposite symbols was that when both independent variables (depression/anxiety) and dietary behavior were included in the model, abnormal dietary behavior positively affected QoL (depression: *β* = 0.012, *p* = 0.001; anxiety: *β* = 0.011, *p* = 0.003) (see Figure 2b). This suggests that individuals with depression and anxiety in unhealthy families may improve their QoL through abnormal dietary behaviors, which masked the negative impact of depression/anxiety on QoL of patients.

**Table 5:** Mediating effect of dietary behavior on the relationship between depression/anxiety and QoL in different family health.

Variables		Family health, *N* = 511				Family unhealth, *N* = 219			
Independent variable	Dependent variable	Effect	*SE*	95%CI		Effect	*SE*	95%CI	
				Lower	Upper			Lower	Upper
Depression	QoL	−0.010***	0.001	−0.011	−0.008	−0.011***	0.002	−0.015	−0.007
Depression	Dietary behavior	0.355***	0.035	0.289	0.424	0.160***	0.042	0.077	0.242
Depression	QoL	−0.009***	0.001	−0.011	−0.007	−0.013***	0.002	−0.017	−0.009
Dietary behavior		−0.001	0.001	−0.003	0.002	0.012***	0.004	0.005	0.019
Anxiety	QoL	−0.012***	0.001	−0.014	−0.010	−0.013***	0.003	−0.019	−0.007
Anxiety	Dietary behavior	0.367***	0.043	0.283	0.450	0.185*	0.056	0.074	0.295
Anxiety	QoL	−0.012***	0.001	−0.014	−0.009	−0.015***	0.003	−0.021	−0.009
Dietary behavior		−0.001	0.001	−0.003	0.001	0.011**	0.004	0.004	0.018

* *p* < 0.05.

** *p* < 0.01.

*** *p* < 0.001.

## 4 Discussion

From the perspective of psychology and behavior, we analyzed the internal mechanisms affecting the QoL of cardiovascular patients and the moderating effect of family health on this mechanism. Through this analysis, we identified psychological factors (depression and anxiety) as important factors affecting QoL. With an increase in depression and anxiety scores, the QoL of patients further decreased. This also confirms the conclusion of previous studies that the QoL of the “psycho-cardiology” population should be considered (48). In this study, abnormal dietary behavior played a mediating role between psychological state (depression/anxiety) and QoL. Our results showed that when we included the mediating variable (i.e., abnormal dietary behavior), the negative effect of depression/anxiety on QoL decreased, suggesting that abnormal dietary behavior did not facilitate, but partially masked the negative effect of depression/anxiety on QoL. This masking effect is more pronounced in patients from unhealthy families.

### 4.1 Analysis of influencing factors on QoL

This study found no significant difference in QoL in terms of gender, ethnicity, BMI, and residence. The EQ-VAS differed from EQ-5D-5L at the highest educational level. The EQ-VAS score increased with higher levels of education; however, the difference in educational level was not significant in the EQ-5D-5L. This may be because EQ-VAS is more susceptible to subjective factors on the day of the questionnaire test than EQ-5D-5L is (49). In this study, the lowest EQ-VAS scores were found in cardiovascular patients with mood disorders, although the EQ-5D-5L scores were not the lowest. This finding suggests that psychological factors may influence how they rate themselves. In the EQ-5D-5L score, patients with only one type of cardiovascular disease had the highest QoL, followed by patients with two or more types of cardiovascular disease, cardiovascular disease with mood disorders, cardiovascular disease combined with other diseases (except mood disorders). This may be because other diseases further affect the QoL of patients with CVD (5). Patients with high depression and anxiety scores generally exhibited lower QoL scores, whereas those with healthy family functioning demonstrated higher QoL scores. The correlation analysis showed that depression, anxiety, dietary behavior, QoL (EQ-5D-5L and EQ-VAS), and family health were significantly correlated. However, negative psychological state (depression and anxiety) and dietary behaviors did not significantly predict EQ-VAS scores in the regression analysis. Therefore, EQ-5D-5L is analyzed as the dependent variable for further discussion.

### 4.2 Mediating effect of dietary behavior

The results of the study support the partially mediating role of abnormal dietary behavior between depression and QoL when both negative mental states and dietary behavior were included. The sign of the indirect effect (depression improved QoL by increasing abnormal dietary behavior) was opposite to the direct effect (depression negatively predicted QoL), which could not be analyzed using the general mediation effect model. According to Wen et al. (47), this situation may be due to the masking effect of abnormal dietary behavior. Depression is the main driving factor of QoL, and its main symptoms include sleep disorders and fatigue, which may lead to deterioration of the patient's physical condition and further decline in QoL (50). However, the major negative impact of depression on QoL was masked in this study. The results of the indirect effects indicate that individuals with higher depression scores were more likely to exhibit abnormal dietary behaviors, and their QoL scores improved rather than decreased. The correlation analysis in this study demonstrated a positive correlation between depression and abnormal dietary behavior, as well as a negative correlation between abnormal dietary behavior and QoL. Why does abnormal dietary behavior increase the QoL when both psychological and dietary behavior variables are included in the mediation effect model? It is well known that abnormal dietary behavior increases the risk for cardiovascular disease especially if the person is anxious and/or depressed (51). In this study, abnormal dietary behavior positively mediated the relationship between depression and QoL and attenuated the negative effect of depression on QoL. Does this suggest that abnormal dietary behavior may be a potential protective factor?

Research has found that consuming highly palatable foods can temporarily suppress negative psychological states (52). According to Heatherton and Baumeister's self-conscious escape theory (53), when depression occurs, they (e.g., emotional eaters) break out of an unpleasant state by eating large amounts of high-sugar, high-fat foods, a tendency to respond to negative emotions through food intake (54, 55). This sense of satisfaction and relief from negative emotions may enable patients with CVD having also depression to believe that their QoL is improved under abnormal dietary behavior patterns, thus reporting higher scores. In fact, the improvement in QoL due to abnormal dietary behavior is temporary, because they immediately experience pleasure by coping with their emotions through binge dietary behavior and forget about real-life problems (56). These behaviors can lead to similar neuroendocrine abnormalities, which consequently induces the accumulation of fat and other risk factors that contribute to cardiovascular disease (57). QoL will be reduced accordingly, even if this effect is covered. Therefore, for patients with CVD, it is necessary to control their dietary behavior rather than improve their QoL by consuming high-calorie foods. In previous studies, poor dietary habits are one of the important factors that reduce the QoL of various population (58). In a study on college students, it was mentioned that the high comorbidity of depression and eating disorders affecting physical activity and exercise motivation can lead to a decline in QoL (59). This is different from the results of this study, possibly because of differences in the study population and study design types. Currently, there is limited research on the relationship between depression, dietary behavior, and QoL in cardiovascular patient populations. Therefore, this study provides a new perspective. Family health moderates the entire mediating effect model.

### 4.3 Moderating effect of family health

Family health moderates the entire mechanism of “negative psychological-dietary behavior-QoL”. In families with healthy family functioning, the mediating role of dietary behavior between negative psychological state (depression/anxiety) and QoL was not significant. Good family health status will give patients great family support and good communication, so that the patient's negative psychological state is improved to avoid emotional overeating and other bad dietary behaviors (60). Simultaneously, the emotional warmth, support, and cohesion of healthy families can improve the patient's ability to recognize the disease and self-care ability, control the intake of diet well, and reduce the deterioration of disease caused by improper diet (61). Family health strategy for mental state and dietary behavior has also made a special emphasis, with a good mental state and reasonable control of dietary intake for the prevention of cardiovascular disease is of great help (62). The results of this study showed that the negative impact of depression and anxiety on QoL was also weakened in healthy families. However, the masking effect of dietary behavior was significantly stronger in unhealthy families, which further explains the positive effect of healthy family functioning. Research shows that unhealthy family circumstances lead to a decline in patients' sense of social and family support, reduced self-efficacy, and aggravation of the patient's negative psychological state (63, 64). Studies have demonstrated that unhealthy families can lead to poor family flexibility, communication, and cohesion, thereby contributing to excessive food consumption (65). When the family communication, family support and other family functions of cardiovascular disease patients with negative psychological status are dysfunctional, the patients will overeat and cannot better face the occurrence and outcome of the disease, ultimately resulting in lower QoL (66).

The results of this study suggest that unhealthy families are more likely to have abnormal dietary behaviors resulting from depression and anxiety, which improve QoL. This may account for reduced behavioral control among unhealthy families. In summary, this discovery underscores the critical importance of comprehending the psychology and eating behavior of cardiovascular patients at the family level. The American Heart Association (AHA) issued a scientific statement on family involvement in adult cardiovascular care in 2022 (67). Engaging family members in care improves person- and family important outcomes, such as the provision of basic care needs (i.e., feeding and hygiene) and assisting with mobilization. There is emerging evidence that this family centered intervention model may improve the management of cardiovascular risk factors. Families can create a supportive environment for improved lifestyles and behaviors that lead to meaningful and lasting changes (68). Thus, family members should pay attention to identifying cardiovascular patients' psychological states in time and develop a reasonable meal plan to prevent the occurrence of emotional coping diets. Family management can provide psychological support and improve unhealthy psychology and negative behavior.

### 4.4 Theoretical implications and practical implications

The findings of this study highlight the interconnected nature of various internal factors influencing the QoL of patients with CVD, rather than acting independently. Therefore, integrated and systematic interventions targeting three key areas are preferable: psychological health (depression and anxiety), dietary behaviors, and family health. This comprehensive approach aims to achieve optimal intervention outcomes and promote the QoL of cardiovascular patients with depression and anxiety, and improve the long-term prognosis of patients. First, our study found that more than half of the cardiovascular patients suffer from depression and anxiety. Early recognition of patients' mental state and formulation of a clinical comprehensive nursing plan for the “psycho-cardiology” population is of great significance in improving patients' QoL. Second, abnormal dietary behaviors may be closely related to the occurrence of depression/anxiety in patients with cardiovascular disease, and mask the negative impact of depression/anxiety on QoL, which needs to be taken seriously. Developing a healthy diet is beneficial for the physical and mental health of people with cardiovascular diseases. It is very important to educate people about the importance of healthy eating and develop simple and feasible eating plans to stimulate patients' positive attitudes. Finally, involving families in cardiovascular care is important for providing psychological support and risk factor management to help patients ease depression and anxiety while adhering to healthy eating habits. The stronger this positive subjective norm, the more it can encourage patients to maintain healthy eating behaviors.

### 4.5 Limitations and future directions

This study encountered several limitations. First, because of its cross-sectional nature, causal relationships between the variables could not be established. Future studies should be conducted using a longitudinal design. In addition, the data sample included in this study was limited. Future studies could attempt to expand the data sample size from multiple information sources to make the test results of the model more convincing. This study discussed the mode of improvement of QoL in patients with CVD and did not exclude patients with CVD combined with other diseases. Further validation of this model in a population with just cardiovascular disease should be considered in the future.

## 5 Conclusion

In conclusion, this study demonstrated the effect of the interaction between negative psychological states (depression and anxiety) and dietary behaviors on QoL in patients with CVD. Meanwhile, abnormal dietary behavior positively mediated the relationship between depression and QoL and attenuated the negative effect of depression on QoL. Family health moderates the entire mediating effect model.

## Data Availability

The raw data supporting the conclusions of this article will be made available by the authors, without undue reservation.
